# Performance of disk diffusion, gradient test, and VITEK 2 for carbapenem susceptibility testing in OXA-48-like carbapenemase-producing *Enterobacterales*: a comparative study

**DOI:** 10.1128/jcm.01893-24

**Published:** 2025-04-16

**Authors:** Cansu Cimen, Philipp Siemer, Janko Sattler, Andreas Voss, Matthijs S. Berends, Axel Hamprecht

**Affiliations:** 1 Institute of Medical Microbiology and Virology, University of Oldenburg 11233, Oldenburg, Germany; 2 Department of Medical Microbiology and Infection Prevention, University Medical Center Groningen, University of Groningen 3647, Groningen, Groningen, The Netherlands; 3 Institute for Medical Microbiology, Immunology and Hygiene, University Hospital Cologne 650804, Cologne, Germany; 4 Department of Machine Learning and Systems Biology, Max Planck Institute of Biochemistry 28311, Martinsried, Germany; 5 Department of Medical Epidemiology, Certe Medical Diagnostics and Advice Foundation 221027 , Groningen, The Netherlands; Johns Hopkins University, Baltimore, Maryland, USA

**Keywords:** carbapenemase, OXA-48-like, carbapenem, antibiotic susceptibility testing, minimum inhibitory concentration, broth microdilution, gradient test, ETEST, disk diffusion, VITEK

## Abstract

This study aimed to compare the performance of disk diffusion, gradient test (ETEST), and VITEK 2 (AST-N223, AST-N428, AST-N432 cards) antibiotic susceptibility testing methods with the reference broth microdilution (BMD) for carbapenem susceptibility in OXA-48-like carbapenemase-producing *Enterobacterales* (CPE). A total of 107 CPE and 142 controls (*Enterobacterales* that do not produce any type of carbapenemases), all molecularly characterized by whole-genome sequencing, were tested for carbapenem susceptibility using BMD and derivative methods. Essential agreement (EA), categorical agreement (CA), major error, very major error, and bias were evaluated. In the OXA-48-like group, resistance frequencies by BMD for ertapenem, imipenem, and meropenem were 86.9%, 12.1%, and 10.3%, respectively. For OXA-48-like CPE, ETEST showed the highest EA among all methods for meropenem (100/107, 93.5%) and ertapenem (99/107, 92.5%), while EA for VITEK 2 cards were <90%. In contrast, for imipenem, VITEK 2 AST-N428 performed best with an EA of 95/105 (90.5%). CA was higher for ertapenem across all methods (93.5%–98.1%) compared to imipenem (59.8%–81.4%) and meropenem (78.8%–95.3%). The highest CA was achieved with ETEST for ertapenem and meropenem, and with VITEK 2 AST-N223 for imipenem. Significant variability was observed across different tests in resistance frequencies, MICs, EA, and CA for the OXA-48-like group. Ertapenem was the most useful carbapenem for detecting resistance in OXA-48-like CPE across all methods. Laboratories should be aware that susceptibility testing of imipenem leads to more erroneous results compared to the other carbapenems when using derivative methods. Additionally, most derivative methods tend to overcall carbapenem resistance in OXA-48-like CPE.

**IMPORTANCE:**

OXA-48-like is the most frequent carbapenemase in western Europe, and both its rapid spread and its challenging-to-detect nature are a particular concern for adequate treatment and infection control purposes. Accurate determination of carbapenem minimal inhibitory concentrations (MICs) is of utmost importance, both for the selection of the best therapy and as a marker for carbapenemase detection. However, the performance of derivative susceptibility testing methods is unclear for OXA-48-like isolates. Our study reports on the varying performance of carbapenem susceptibility testing by disk diffusion, gradient test (ETEST), and VITEK 2 in OXA-48-like-producing *Enterobacterales*. The results of the present study can help to inform about the limitations of current susceptibility testing methods and serve to improve MIC determination in these challenging isolates.

## INTRODUCTION

Carbapenemase-producing *Enterobacterales* (CPE) have emerged as a significant threat to healthcare worldwide (1). Carbapenemase-encoding genes are often located on plasmids, and this makes them highly transmissible and creates a substantial burden for patient management (1–4).

Notably, OXA-48-like carbapenemases are the most frequent group in western Europe (1, 5). The rapid spread of these carbapenemases is a particular concern as they are challenging to detect, and this situation potentially leads to ineffective treatment and inadequate infection control measures (5, 6). Due to their weak hydrolytic activity, bacteria producing OXA-48-like carbapenemases often exhibit only slightly elevated minimum inhibitory concentrations (MICs) for carbapenems, which might lead to categorization as susceptible (S) or susceptible increased exposure (I) (6). High MICs for piperacillin-tazobactam and temocillin, in combination with elevated carbapenem MICs, are indicative of OXA-48-like enzyme production, but relying solely on these parameters has limitations, especially in challenging species such as *Proteus mirabilis* (6–9). Furthermore, unlike metallo-beta-lactamases (MBLs) or *Klebsiella pneumoniae* carbapenemases (KPCs), no phenotypic inhibitor tests are currently available to identify OXA-48-like enzymes (4, 5, 10).

Immunochromatographic tests and PCR are reliable confirmatory methods for detecting OXA-48-like enzymes (11). However, accurate carbapenem susceptibility testing and reliable MIC values are the first steps to select isolates for confirmatory testing. While broth microdilution (BMD) remains the gold standard for determining carbapenem MICs, its extensive hands-on time makes it impractical for diagnostic laboratories. Thus, numerous derivative testing methods are available. Previous studies have reported inconsistent results between different antimicrobial susceptibility testing (AST) methods for carbapenem susceptibility testing (12–19), but data specifically addressing OXA-48 CPE are limited.

Therefore, we conducted a comparative study to evaluate the performance of disk diffusion (DD), gradient test (ETEST), and VITEK 2 in comparison with BMD as the reference method for OXA-48-like CPE.

## MATERIALS AND METHODS

### Study design and clinical isolates

This laboratory-based comparative study comprised 249 non-duplicate clinical isolates (OXA-48 = 75, OXA-181 = 13, OXA-244 = 12, OXA-162 = 7; control = 142), collected from 2012 to 2021 at the University Hospital Cologne and Klinikum Oldenburg (Table 1). Control isolates refer to *Enterobacterales* that do not produce any type of carbapenemases. The isolates were grown from different clinical samples, including urine, blood, tissues, wound swabs, other body fluids, rectal swabs, and stool. All isolates had been molecularly characterized by whole-genome sequencing (WGS) as previously described (20, 21).

**TABLE 1:** Isolate distribution by species and corresponding molecular characterization of beta-lactamase gene content

Molecular category	Species							
	*Citrobacter* spp.	*Enterobacter* spp.	*Escherichia coli*	*Klebsiella* spp.	*Proteus mirabilis*	*Raoultella ornithinolytica*	*Serratia marcescens*	Total
OXA-48-like	20	9	37	35	3	1	2	107
*bla*_OXA-48_	14	8	15	32	3	1	2	75
*bla*_OXA-162_	5	1	1	0	0	0	0	7
*bla*_OXA-181_	1	0	10	2	0	0	0	13
*bla*_OXA-244_	0	0	11	1	0	0	0	12
ESBL-positive	15	4	22	31	2	0	0	74 (69.2%)
ESBL-negative	5	5	15	4	1	1	2	33 (30.8%)
Control	8	33	24	64	5	0	8	142
ESBL								
CTX-M-1 group	3	0	13	21	0	0	1	38
CTX-M-9 group	0	1	1	4	0	0	0	6
Other ESBLs	0	0	0	12	2	0	0	14
AmpC								
*bla*_ACT-7_	0	12	0	0	0	0	0	12
*bla*_CMY-2_	3	0	4	1	0	0	0	8
Other AmpCs	5	19	4	4	3	0	6	41
Other beta- lactamases	5	2	16	43	3	0	1	70

The isolates were stored at −80°C and subcultured twice on 5% sheep blood agar (Becton Dickinson GmbH, Heidelberg, Germany) before susceptibility testing. Identification of isolates was verified using matrix-assisted laser desorption-ionization-time of flight mass spectrometry (MALDI-TOF MS) (Biotyper, Bruker, Bremen, Germany).

### AST

**Fig 1:**
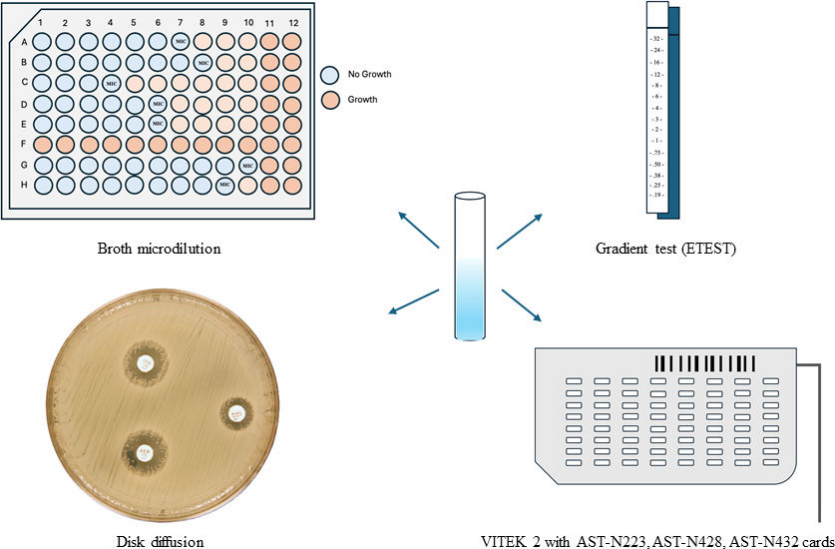
Antibiotic susceptibility testing methods evaluated in this study.

All assays were performed using the same 0.5 MacFarland inoculum (Fig. 1). Results were interpreted according to the European Committee on Antimicrobial Susceptibility Testing (EUCAST) clinical breakpoints (v14.0) (22). DD and ETEST were visually read following the manufacturer’s guidelines, with the researcher blinded to the molecular characteristics of the isolates. MIC calling ranges for each method are provided in Table S1. *Escherichia coli* (ATCC 25922), *Pseudomonas aeruginosa* (ATCC 27853), and *K. pneumoniae* (ATCC 700603, ATCC BAA-2814) were used as quality control strains for AST.

In case of discrepancies between the reference method and a derivative method (>2 dilutions for an MIC method or categorical discrepancy for DD), the isolate was retested to exclude any technical error before the final analysis.

### BMD

Custom-manufactured BMD assays (Micronaut-S, Bruker Daltonics, Bremen, Germany) were used for BMD and were visually read with an inverted mirror after incubation at 36°C for 18–24 h.

### DD

DD was performed according to EUCAST, using meropenem 10 µg, ertapenem 10 µg, and imipenem 10 µg disks on Mueller-Hinton agar (MHA); all obtained from Oxoid, Basingstoke, UK.

### Gradient test

Carbapenem gradient tests (ETEST, bioMérieux, Marcy l’Etoile, France) were placed on MHA (bioMérieux) inoculated with bacterial suspension and read after incubation at 36°C for 18–20 h.

### VITEK 2

VITEK 2 testing using AST-N223, AST-N428, and AST-N432 cards (bioMérieux) was performed following the manufacturer’s recommendations with VITEK 2 software v9.03.

Some isolates had irregular growth, and therefore no MIC values could be obtained by VITEK 2. Additionally, production of the VITEK-AST N223 card was discontinued at the time of retesting, so some isolates could not be assessed with this card.

### WGS

DNA was extracted using the DNeasy UltraClean Microbial Kit (Qiagen, Hilden, Germany). WGS was performed for all isolates on the Illumina platform (Illumina, San Diego, USA). Raw reads were *de novo* assembled using Spades v3.14.1, and resistance gene content was determined with the ResFinder database using ABRicate 1.0.1 (23, 24).

### Statistical analysis

BMD served as the reference for comparing methods. Essential agreement (EA), categorical agreement (CA), major error (ME), very major error (VME), and bias were evaluated according to ISO guidelines (25, 26). Bias was assessed to determine whether deviations from the reference method were significantly skewed or predominantly in one direction (25). Acceptable performance was based on ISO thresholds: ≥90% for EA or CA, ≤3% for ME or VME, and <30% for bias (25). Mann-Whitney *U* test and Pearson’s *χ*^2^ test were employed for statistical comparisons. A *P* value <0.05 was considered significant.

## RESULTS

Among the OXA-48-like CPE, 69.2% harbored an additional ESBL gene. The control isolates exhibited other resistance mechanisms, including ESBL, AmpC, and other beta-lactamases, with 135/142 showing MICs above the EUCAST screening cut-off for at least one carbapenem (Tables 1 and 2).

**TABLE 2:** MIC ranges (including MIC_50_ and MIC_90_) and categorization of susceptibility results of the isolates^*e*^^,^^*f*^

Antibiotic	Method	MIC distribution						Categorical distribution											
		OXA-48-like			Controls			OXA-48-like						Controls					
		MIC_50_	MIC_90_	*P^b^*	MIC_50_	MIC_90_	*P* ^*b*^	S	I	R	%R	Total^*d*^	*P* ^*c*^	S	I	R	%R	Total^*d*^	*P^c^*
Ertapenem	Broth microdilution	2	16	*–*	2	16	*–*	14	–	93	86.9	107	*–*	37	–	105	73.9	142	*–*
	Disk diffusion	–	–	*–*	–	–	*–*	7	–	100	93.5	107	0.168	24	–	118	83.1	142	0.082
	Gradient test (ETEST)	2	≥32	0.974	2	≥32	0.625	16	–	91	85.0	107	0.843	35	–	107	75.4	142	0.891
	VITEK 2 AST-N223	4	≥8	**<0.001**	4	≥8	**0.006**	10	–	94	90.4	104	0.658	23	–	101	81.5	124	0.352
	VITEK 2 AST-N428	2	≥8	**0.024**	4	≥8	0.091	12	–	94	88.7	106	0.834	36	–	106	74.6	142	1.00
** **	VITEK 2 AST-N432	2	≥8	**0.006**	4	≥8	**0.021**	13	–	94	87.9	107	1.00	31	–	111	78.2	142	0.486
Imipenem^*a*^	Broth microdilution	2	8	*–*	0.5	4	*–*	83	11	13	12.1	107	*–*	125	7	10	7.0	142	*–*
	Disk diffusion	–	–	*–*	–	–	*–*	52	30	25	23.4	107	**0.049**	118	10	14	9.9	142	0.522
	Gradient test (ETEST)	1	16	0.924	1	8	0.062	73	13	21	19.6	107	0.190	115	9	18	12.7	142	0.163
	VITEK 2 AST-N223	2	≥16	0.065	1	8	0.448	74	9	19	18.6	102	0.108	103	3	13	10.9	119	0.660
	VITEK 2 AST-N428	1	4	0.813	1	4	0.785	75	22	8	7.6	105	0.357	125	7	8	5.7	140	0.807
Meropenem	Broth microdilution	1	16	*–*	0.5	8	*–*	84	12	11	10.3	107	*–*	112	26	4	2.8	142	*–*
	Disk diffusion	–	–	*–*	–	–	*–*	67	28	12	11.2	107	1.00	99	32	11	7.7	142	0.111
	Gradient test (ETEST)	1	≥32	0.216	0.5	8	0.589	86	9	12	11.2	107	1.00	111	18	13	9.2	142	**0.045**
	VITEK 2 AST-N223	2	≥16	**0.007**	1	*4*	0.240	71	20	13	12.5	104	0.828	96	23	5	4.0	124	1.00
	VITEK 2 AST-N428	2	≥16	**0.009**	1	4	0.813	74	20	13	12.1	107	0.828	116	21	5	3.5	142	1.00
** **	VITEK 2 AST-N432	2	≥16	**0.004**	1	4	0.531	67	29	11	10.3	107	1.00	114	23	5	3.5	142	1.00

^*a*^ Imipenem is not available on the AST-N432 card.

^*b*^ Comparison with MIC distribution of BMD (Mann-Whitney *U* test).

^*c*^ Comparison with R frequency of BMD (Pearson’s *χ*^2^ test).

^*d*^ Numbers are given based on the determined MIC from the respective test. While results were obtained for all isolates by BMD, ETEST, and DD, VITEK 2 MICs for some isolates could not be determined by N223/N428 cards.

^*e*^ EUCAST clinical breakpoints: ertapenem, MIC: ≤0.5 and >0.5, zone diameter: ≥23 and <23; imipenem, MIC: ≤2 and >4, zone diameter: ≥22 and <19; meropenem, MIC: ≤2 and >8, zone diameter: ≥22 and <16 (22).

^*f*^ S, susceptible; I, susceptible, increased exposure; R, resistant. "-" represents not applicable (N/A). Bolded when *P*<0.05.

The distribution of MICs and susceptibility categorization of the 249 isolates are summarized in Table 2. The highest frequency of resistance was observed for ertapenem in both the OXA-48-like and control groups. In the OXA-48-like group, resistance frequencies by BMD were 86.9% for ertapenem, 12.1% for imipenem, and 10.3% for meropenem (Table 2). When compared with the reference method, derivative methods tended to overcall resistance, except for ETEST for ertapenem, VITEK 2 AST-N428 for imipenem, and VITEK 2 AST-N432 for meropenem (Table 2). A significant difference (*P* < 0.05) in resistance frequency was observed only for imipenem by DD in the OXA-48-like group. In addition to EUCAST, susceptibility categorization of the isolates was also presented when CLSI breakpoints were applied (Table S2).

The MIC_50_/MIC_90_ values determined by different tests showed substantial variability when compared with the reference method BMD. Statistically significant differences (*P* < 0.05) were observed for the following: all VITEK 2 cards for ertapenem and meropenem in the OXA-48-like group, VITEK 2 AST-N223 and VITEK 2 AST-N432 for ertapenem in the control group. In these cases, the MIC values determined by VITEK 2 were higher than those determined by BMD. Tables S3 to S5 present the detailed and comparative MIC distributions for each test. Tables comparing the MIC value distributions obtained by BMD and zone diameters by DD for each antibiotic are available in Table S6.

The EA for carbapenems was <90% for most methods in both the OXA-48-like group and the control group (Table 3). In the OXA-48-like group, ETEST showed the highest EA for meropenem (93.5%), followed by ertapenem (92.5%), while EA was <90% for all VITEK 2 cards. In contrast, the highest EA for imipenem (90.5%) was observed with VITEK 2 AST-N428. Bias was >30% for ertapenem and meropenem with all VITEK cards in the OXA-48-like group and for ertapenem with VITEK 2 AST-N223 and VITEK 2 AST-N432 cards in the control group, pointing out a tendency to overestimate MICs for these antibiotics.

**TABLE 3:** Comparison of different antimicrobial susceptibility testing methods with BMD for carbapenems^*e*^^,*f*^

Antibiotic	Method	OXA-48-like					Controls				
		EA (%)	Bias^*b*^ (%)	CA (%)	ME^*c*^ (%)	VME^*d*^ (%)	EA (%)	Bias (%)	CA (%)	ME (%)	VME (%)
Ertapenem	Broth microdilution	–	–	–	–	–	–	–	–	-–	–
	Disk diffusion	–	–	100/107 (**93.5**)	7/14 (50.0)	0 (0.0)	–	–	129/142 (**90.8**)	13/37 (35.1)	0 (0.0)
	Gradient test (ETEST)	99/107 (**92.5**)	2.2	105/107 (**98.1**)	0 (0.0)	2/93 (2.2)	127/142 (89.4)	8.1	126/142 (88.7)	9/37 (24.3)	7/105 (6.7)
	VITEK 2 AST-N223	81/104 (77.9)	70.1	101/104 (**97.1**)	3/13 (23.1)	0 (0.0)	99/124 (79.8)	46.6	103/124 (83.1)	14/30 (46.7)	7/94 (7.4)
	VITEK 2 AST-N428	93/106 (87.7)	38.8	102/106 (**96.2**)	3/14 (21.4)	1/92 (1.1)	106/142 (74.6)	25.8	115/142 (81.0)	14/37 (37.8)	13/105 (12.4)
** **	VITEK 2 AST-N432	88/107 (82.2)	45.4	102/107 (**95.3**)	3/14 (21.4)	2/93 (2.2)	105/142 (73.9)	31.5	118/142 (83.1)	15/37 (40.5)	9/105 (8.6)
Imipenem^*a*^	Broth microdilution	–	–	–	–	–	–	–	–	–	–
	Disk diffusion	–	–	64/107 (59.8)	4/83 (4.8)	0 (0.0)	–	–	129/142 (**90.8**)	0 (0.0)	0 (0.0)
	Gradient test (ETEST)	92/107 (86.0)	6.8	84/107 (78.5)	5/83 (6.0)	0 (0.0)	125/142 (88.0)	29.6	127/142 (89.4)	3/125 (2.4)	0 (0.0)
	VITEK 2 AST-N223	86/102 (84.3)	24.7	83/102 (81.4)	5/81 (6.2)	0 (0.0)	107/119 (89.9)	7.5	109/119 (**91.6**)	2/104 (1.9)	2/10 (20.0)
	VITEK 2 AST-N428	95/105 (**90.5**)	−4.3	81/105 (77.1)	2/81 (2.5)	1/13 (7.7)	119/140 (85.0)	−14.4	127/140 (**90.7**)	2/123 (1.6)	3/10 (30.0)
Meropenem	Broth microdilution	–	–	–	–	–	–	–	–	–	–
	Disk diffusion	–	–	87/107 (81.3)	0 (0.0)	0 (0.0)	–	–	120/142 (84.5)	0 (0.0)	0 (0.0)
	Gradient test (ETEST)	100/107 (**93.5**)	−16.2	102/107 (**95.3**)	0 (0.0)	0 (0.0)	131/142 (**92.3**)	16	128/142 (**90.1**)	0 (0.0)	0 (0.0)
	VITEK 2 AST-N223	79/107 (76.0)	30.5	82/104 (78.8)	0 (0.0)	0 (0.0)	110/124 (88.7)	3.3	114/124 (**91.9**)	0 (0.0)	0 (0.0)
	VITEK 2 AST-N428	88/107 (82.2)	33.5	89/107 (83.2)	0 (0.0)	0 (0.0)	126/142 (88.7)	−14.7	133/142 (**93.7**)	0 (0.0)	0 (0.0)
	VITEK 2 AST-N432	78/107 (72.9)	34.6	82/107 (76.6)	0 (0.0)	0 (0.0)	125/142 (88.0)	−4.0	133/142 (**93.7**)	0 (0.0)	0 (0.0)

^*a*^ Imipenem is not available on the AST-N432 card.

^*b*^ Bias is the difference between the percentage of test results that are greater than the reference method and the percentage of results that are smaller. A bias value >30% indicates that the MIC determined by the respective test is predominantly higher than the MIC determined by the reference method, suggesting an overestimation of resistance compared to BMD.

^*c*^ ME is the proportion of false-resistant results among the total number of susceptible isolates as determined by the reference method.

^*d*^ VME is the proportion of false susceptible results among the total number of resistant isolates as determined by the reference method.

^*e*^ EA (%) and CA (%) bolded when ≥90%. "-" represents not applicable (N/A).

^*f*^ While results were obtained for all isolates by BMD, ETEST, and DD, VITEK 2 MICs for some isolates could not be determined by N223/N428 cards.

In the OXA-48-like group, CA for ertapenem was consistently higher (93.5%–98.1%) across all methods compared to imipenem (59.8%–81.4%) and meropenem (78.8%–95.3%) (Table 3). The highest CA for ertapenem and meropenem was determined using ETEST, whereas the highest CA for imipenem was reported with the VITEK 2 AST-N223 card. The highest ME frequencies were observed for ertapenem (21.4%–50.0%). VME values ≥3% were not recorded for ertapenem; however, a VME of 7.7% was observed for imipenem with the VITEK 2 AST-N428 card. No ME or VME for meropenem was recorded in either group.

Overall (OXA-48-like group and control group) comparison regarding EA, CA, bias, and error frequencies of different AST methods with BMD for carbapenems is presented in Table S7. A comparison of the EA, CA, and error frequencies for each method between the two groups is also reported in Table S8.

In our data collection, the EUCAST screening cut-off values (MIC >0.125 mg/L for meropenem and ertapenem; zone diameter <28 mm for meropenem and <25 mm for ertapenem) for carbapenemase detection exhibited high sensitivity but low specificity for both meropenem (95.3%–98.1%/16.2%–24.6%) and ertapenem (97.2%–98.1%/4.2%–7.7%) (27). Isolates that were below this screening cut-off by BMD were *E. coli* (*n* = 3) for meropenem and *P. mirabilis* (*n* = 2) for ertapenem. Table 4 summarizes the sensitivity, specificity, and the species that were overlooked by each test.

**TABLE 4:** Sensitivity and specificity of the EUCAST screening cut-off values for detecting carbapenemases in OXA-48-like-producing isolates of the study^*c*^

Antibiotic	Method	Sensitivity (%)	Specificity (%)	OXA-48-like CPEs below the EUCAST screening cut-off
Meropenem^*a*^	Broth microdilution	104/107 (97.2)	24/142 (16.9)	*E. coli* (*n* = 3)
	Gradient test (ETEST)	102/107 (95.3)	23/142 (16.2)	*E. coli* (*n* = 2), *P. mirabilis* (*n* = 3)
	Disk diffusion	105/107 (98.1)	35/142 (24.6)	*P. mirabilis* (*n* = 2)
Ertapenem^*b*^	Broth microdilution	105/107 (98.1)	7/142 (4.9)	*P. mirabilis* (*n* = 2)
	Gradient test (ETEST)	104/107 (97.2)	6/142 (4.2)	*P. mirabilis* (*n* = 3)
	VITEK 2 AST-N428	103/106 (97.2)	11/142 (7.7)	*P. mirabilis* (*n* = 3)
	VITEK 2 AST-N432	104/107 (97.2)	9/142 (6.3)	*P. mirabilis* (*n* = 3)
	Disk diffusion	104/107 (97.2)	13/142 (9.2)	*P. mirabilis* (*n* = 3)

^*a*^ Not evaluable for VITEK 2 cards, the lowest meropenem MIC value ≤0.25 mg/L.

^*b*^ Not evaluable for VITEK 2 AST-N223 card, the lowest ertapenem MIC value ≤0.5 mg/L.

^*c*^ Screening cut-off for meropenem: MIC >0.125 mg/L, zone diameter, >28 mm; screening cut-off for ertapenem: MIC >0.125 mg/L, zone diameter, >25 mm (27).

## DISCUSSION

We analyzed carbapenem susceptibility in a large number of molecularly characterized OXA-48-like and control group isolates with a wide MIC distribution by comparing the performance of different methods with BMD as the reference. Substantial variability was observed between derivative test methods in carbapenem resistance, MICs, EA, CA, error frequencies, and bias in both groups.

Despite the high numbers of infections with OXA-48-producing isolates and the challenges of susceptibility testing, there are currently only a few studies reporting the performance of different tests in OXA-48-like CPEs (17, 19). Both previous studies analyzed a single or few species and isolates (*n* = 6 and *n* = 82). In the present study, acceptable EA according to ISO criteria (≥90%) was achieved only by ETEST for ertapenem and meropenem, and by VITEK 2 AST N428 for imipenem. Non-satisfying EA with BMD between ETEST and VITEK 2 systems has been previously documented in KPC-producing, MBL-producing, and OXA-48-like-producing *Enterobacterales* (14, 17, 28). A recent study on OXA-48-like *Enterobacterales* also observed significant discrepancies between the carbapenem MICs obtained by ETEST and VITEK 2; however, this study included only isolates of *K. pneumoniae*, and no molecular characterization of OXA-48 variants was performed (19).

Reliable MIC determination is indispensable in clinical practice, especially since recent guidelines warn against using carbapenem-based combination therapy for infections with carbapenem-resistant *Enterobacterales* unless high-dose extended-infusion meropenem is employed and the MIC is ≤8 mg/L (29). In a cohort study on OXA-48-producing *K. pneumoniae* infections, carbapenem-containing monotherapy was superior to non-carbapenem monotherapy in clinical response by day 14 and all-cause mortality by day 30 (30). This was observed especially when a carbapenem MIC within the susceptible range (≤8 mg/L) was recorded which shows the importance of MIC values in treatment decisions (30). In our study, 96 isolates had a meropenem MIC of ≤8 mg/L by BMD. Among these, an MIC value >8 mg/L was observed in only one isolate by ETEST, while VITEK 2 AST-N223 and VITEK 2 AST-N428 yielded MIC values >8 mg/L in two isolates, with one of these isolates being the same as the one identified by ETEST. Therefore, it should be noted that with derivative methods, treatment with carbapenem-containing regimens may be missed due to an overestimation of resistance. In case a carbapenem-containing regimen is indicated, MICs should be rechecked with a second method, preferably with BMD. On the other hand, the consistent reporting of meropenem MICs >8 mg/L by both ETEST and VITEK 2 for all isolates with MICs >8 mg/L by BMD is reassuring. This suggests that these methods slightly overestimate resistance rather than underestimate it at this breakpoint and would not lead to the use of inappropriate meropenem therapy.

For imipenem, no acceptable CA was observed with DD, ETEST, or VITEK 2 AST-N223 card, consistent with findings from a recent study that compared carbapenem MICs of OXA-48-like *K. pneumoniae* using ETEST, VITEK 2 (AST-N255), Sensititre, and MicroScan (19). The lowest CA (59.8%) was observed with DD for imipenem in the OXA-48-like group. This result suggests that an alternative test method is needed to ensure accurate imipenem susceptibility determination in OXA-48-like CPE, which was not the case for the control group in our study. Furthermore, the CA levels for meropenem with DD and VITEK 2 cards were all <90%, consistent with findings from a study on OXA-48-like CPE with reduced susceptibility to meropenem (17). In our study, DD yielded a CA of 81.3% for meropenem, compared to 62% in the previous study (17). Using three different VITEK 2 cards, CA ranged from 76.6% to 83.2% in our study, while the aforementioned study reported a CA of 69.5% with VITEK 2 (AST-N230, AST-N218, and AST-N209) (17).

ME and VME have been associated with worse patient outcomes due to inappropriate targeted treatment, which indicates the importance of reliable AST (31). High (>3%) and varying ME and VME frequencies for commercial methods were reported in KPC-producing and MBL-producing *Enterobacterales* (14, 28, 31). In OXA-48-like-producing *Enterobacterales,* a study found no VME for meropenem using DD and VITEK 2, similar to our findings (17). However, that study reported a high ME frequency of 7% with DD , while we did not observe any ME for meropenem in our study. The observed differences could be due to the low number of OXA-48 in the previous study (*n* = 6), the use of different brands of disks used for DD, and different VITEK 2 cards compared to the present study (17).

Determining carbapenem MIC values is necessary for treatment decisions but also useful to distinguish whether carbapenem-resistant *Enterobacterales* produce carbapenemases or to select isolates for further testing (32). EUCAST provides screening cut-off values to predict carbapenemase production (27). However, the rapid identification of a carbapenemase remains important to initiate adequate treatment, e.g., with novel beta-lactam/beta-lactamase inhibitor combinations (32, 33). Several methods can be used to identify the carbapenemase, e.g., PCR or immunochromatographic assays (32). In our data collection, the detection of presumed OXA-48-like carbapenemase producers based on meropenem and ertapenem EUCAST screening cut-off values achieved high sensitivity, but very low specificity. It was remarkable that not all CPE isolates had MICs (determined by BMD) above the EUCAST screening cut-off values for both meropenem and ertapenem. Three *E. coli* isolates had a meropenem MIC of 0.125 mg/L, and two *P. mirabilis* isolates had an ertapenem MIC of 0.06 mg/L. It has previously been shown that CPE can have carbapenem MICs below the EUCAST screening cut-off values, typically isolates producing OXA-48-like enzymes and/or carbapenemase-producing *P. mirabilis* (8, 9). The OXA-48-like-producing isolates with MICs below the EUCAST screening breakpoint in our data set have carbapenem MICs above the EUCAST epidemiological cut-off (ECOFF) values for *E. coli* and *P. mirabilis* and would thus still be identified as non-wildtype. Therefore, the application of species-specific carbapenem screening cut-off values instead of a general *Enterobacterales* cut-off could improve the sensitivity of carbapenemase detection. The low specificity of the EUCAST screening cut-off values was likely due to our challenge collection, with control group isolates that were selected for elevated carbapenem MICs and/or production of different resistance mechanisms such as ESBL (58/142) and AmpC (61/142). It is well known that carbapenem resistance can also be caused by ESBL production or AmpC derepression/overproduction in addition to decreased permeability (32, 34). Our challenge collection is not representative of a typical non-CPE population in most regions. Likely, all methods would have shown a better performance when challenged with a higher number of “standard” isolates. Another point to highlight is that not all automated systems have the necessary low dilutions to apply screening cut-off values, as was the case in our study with all VITEK cards for meropenem and VITEK 2 AST-223 for ertapenem. The new VITEK 2 AST cards (AST-N428 and AST-N432) feature a lower MIC range for ertapenem (including ≤0.12 mg/L), compared to the older AST-N223 card. This allows more accurate AST results and facilitates the application of EUCAST screening cut-off values for carbapenemase detection.

There were some limitations to this study, e.g., using a commercial BMD assay as the gold standard, testing each isolate only once with each method, and employing a single reader for BMD, DD, and ETEST. For DD, disks from a single manufacturer were used. However, we chose this manufacturer because the high and reproducible quality was demonstrated in the EUCAST evaluation study (35). The performance of AST methods may differ by varying expression of resistance genes, inoculum size, incubation time and conditions, media, and disk types used, as well as between different strains (36). However, we employed a large collection of molecularly well-characterized strains covering a wide range of MICs. Additionally, results can be better compared, as the same inoculum was used for all assays. Nevertheless, our findings should be further investigated in other settings with strains of diverse origins.

In conclusion, substantial variability in the carbapenem MICs, CA, EA, error frequencies, and bias for OXA-48-like CPE and control isolates were observed across the examined methods. Laboratories should be aware of the limitations of the test they use, e.g., especially the low CA for imipenem with most methods, which should preferably be tested by BMD or could be blinded on the report. Additionally, laboratories should be aware that most methods tend to overcall resistance for carbapenems. Based on its performance across three carbapenems, ETEST showed the highest overall performance in determining the MIC and susceptibility in OXA-48-like CPE, with low bias and error frequencies.

## SUPPLEMENTAL MATERIAL

Tables S1 to S8 jcm.01893-24-s0001.xlsx MIC calling ranges of the tests and detailed and comparative MIC distributions and disk diffusion zone diameters of the isolates.

ASM does not own the copyrights to Supplemental Material that may be linked to, or accessed through, an article. The authors have granted ASM a non-exclusive, world-wide license to publish the Supplemental Material files. Please contact the corresponding author directly for reuse.
